# An artificial intelligence model for the radiographic diagnosis of osteoarthritis of the temporomandibular joint

**DOI:** 10.1038/s41598-023-43277-6

**Published:** 2023-09-25

**Authors:** Wael M. Talaat, Shishir Shetty, Saad Al Bayatti, Sameh Talaat, Louloua Mourad, Sunaina Shetty, Ahmed Kaboudan

**Affiliations:** 1 Department of Oral and Craniofacial Health Sciences, College of Dental Medicine, University of Sharjah, Sharjah, 27272 UAE; 2 Research Institute for Medical and Health Sciences, University of Sharjah, Sharjah, 27272 UAE; 3 Department of Oral and Maxillofacial Surgery, Faculty of Dentistry, Suez Canal University, Ismailia, Egypt; 4 Chair, Department of Oral and Craniofacial Health Sciences, College of Dental Medicine, University of Sharjah, Sharjah, UAE; 5 Department of Orthodontics, Future University in Egypt, Cairo, Egypt; 6 Department of Oral Technology, University Clinic, Bonn, Germany; 7 Department of Oral and Maxillofacial Surgery, Faculty of Dentistry, Beirut Arab University, Tripoli, Lebanon; 8 Department of Restorative and Preventive Dentistry, College of Dental Medicine, University of Sharjah, Sharjah, 27272 UAE; 9 Department of Computer Science, Shorouk Academy, El Shorouk, Egypt; 10 Interdisciplinary AI Hub, Future University in Egypt, Cairo, Egypt; 11 DigiBrain4 Inc, Chicago, USA

**Keywords:** Diseases, Medical research, Rheumatology, Signs and symptoms

## Abstract

The interpretation of the signs of Temporomandibular joint (TMJ) osteoarthritis on cone-beam computed tomography (CBCT) is highly subjective that hinders the diagnostic process. The objectives of this study were to develop and test the performance of an artificial intelligence (AI) model for the diagnosis of TMJ osteoarthritis from CBCT. A total of 2737 CBCT images from 943 patients were used for the training and validation of the AI model. The model was based on a single convolutional network while object detection was achieved using a single regression model. Two experienced evaluators performed a Diagnostic Criteria for Temporomandibular Disorders (DC/TMD)-based assessment to generate a separate model-testing set of 350 images in which the concluded diagnosis was considered the golden reference. The diagnostic performance of the model was then compared to an experienced oral radiologist. The AI diagnosis showed statistically higher agreement with the golden reference compared to the radiologist. Cohen’s kappa showed statistically significant differences in the agreement between the AI and the radiologist with the golden reference for the diagnosis of all signs collectively (*P* = 0.0079) and for subcortical cysts (*P* = 0.0214). AI is expected to eliminate the subjectivity associated with the human interpretation and expedite the diagnostic process of TMJ osteoarthritis.

## Introduction

Osteoarthritis is a chronic inflammatory disease that has serious consequences affecting the quality of life. This common disease leads to pain and dysfunction that negatively impact the quality of sleep and the ability to work^1^. The consistently increasing prevalence of osteoarthritis is a major cause of concern to the health authorities especially considering the complexities and the high cost of treatment. Osteoarthritis was ranked as the second cause of the “increase in years lived with disability” following diabetes^2,3^. The etiology of TMJ osteoarthritis is complex and multifactorial, and the disorder is characterized by a progressive cartilage and bone degradation and remodeling^4^. The inflammatory arthritic condition has been attributed to the increased level of inflammatory cytokines that is regulated by monocyte chemoattraction and the decreased biomechanical properties of the disc leading to loss of the adaptive capacity of the TMJ^5^. It has been shown that the escalated turnover of the subchondral bone accounts for the initiation and progression of osteoarthritis. The increased turnover has been attributed to the increased expression of the genes responsible for the osteoclastic activity and increased osteoclastogenesis^6^.

The management of temporomandibular disorders (TMD) often suffers from the inconsistency and controversies that surrounds the diagnostic and treatment protocols and the lack of evidence related to the contemporary management principles. This often results in delays in the diagnosis and progression of the disorders to more advanced forms^7,8^. In addition, TMD-related referred pain obscures the origin of TMD pain, as the pain spreads to other locations that are distant from the joint and the related muscles which aggravates the complexity of the diagnostic procedure^9^. In spite of the remarkable value of the early diagnosis of TMD in stopping the progression to the severe forms of these disorders, however the process is hindered by the mentioned complexities as well as the subjectivity of the diagnosis that was evident in several studies^3,10–12^.

A reliable diagnostic protocol for TMD was published in 2009 as the Research Diagnostic Criteria for Temporomandibular Disorders (RDC/TMD)^13^, and was updated in 2014 as the Diagnostic Criteria for Temporomandibular Disorders (DC/TMD)^14^. The diagnostic criteria integrates diagnostic decision trees that rely on the clinical and radiographic diagnostic criteria (Axis-I) and the psychosocial condition and pain-related disabilities (Axis-II). The reliability of the DC/TMD for the diagnoses of the different TMD classes has been reported as excellent^15^. The DC/TMD defines the degenerative joint disease as a disorder that involves degeneration of the articular tissues combined with osseous changes on the condyle and the articular eminence that are detected on computed tomography (CT) and CBCT in the form of subchondral (subcortical) cyst, erosions, sclerosis, flattening and osteophyte^16^. The interpretation of these signs is highly subjective and there is an urgent need to eliminate this subjectivity to enhance the diagnosis of TMJ osteoarthritis^17,18^.

Deep learning is a category of AI that has been successfully utilized to facilitate diagnosis and enhance decision making in clinical dentistry. The dominant AI model that is used in medical imaging is the neural network applications. When applied to radiomic data, deep learning can identify the complex patterns within the dataset and thus can lead to the objective diagnosis of TMJ osteoarthritis. The AI-supported clinical decision making in the field of TMD may significantly reduce the percentage of cases that progress to the complicated forms of TMD by allowing early diagnosis^19^. This study aimed to develop and test the performance of an AI model based on neural networks for the diagnosis of TMJ osteoarthritis from CBCT. We hypothesized that there is a high degree of conformity between the expert clinicians and AI in diagnosing TMJ osteoarthritis.

## Methodology

**Figure 1:**
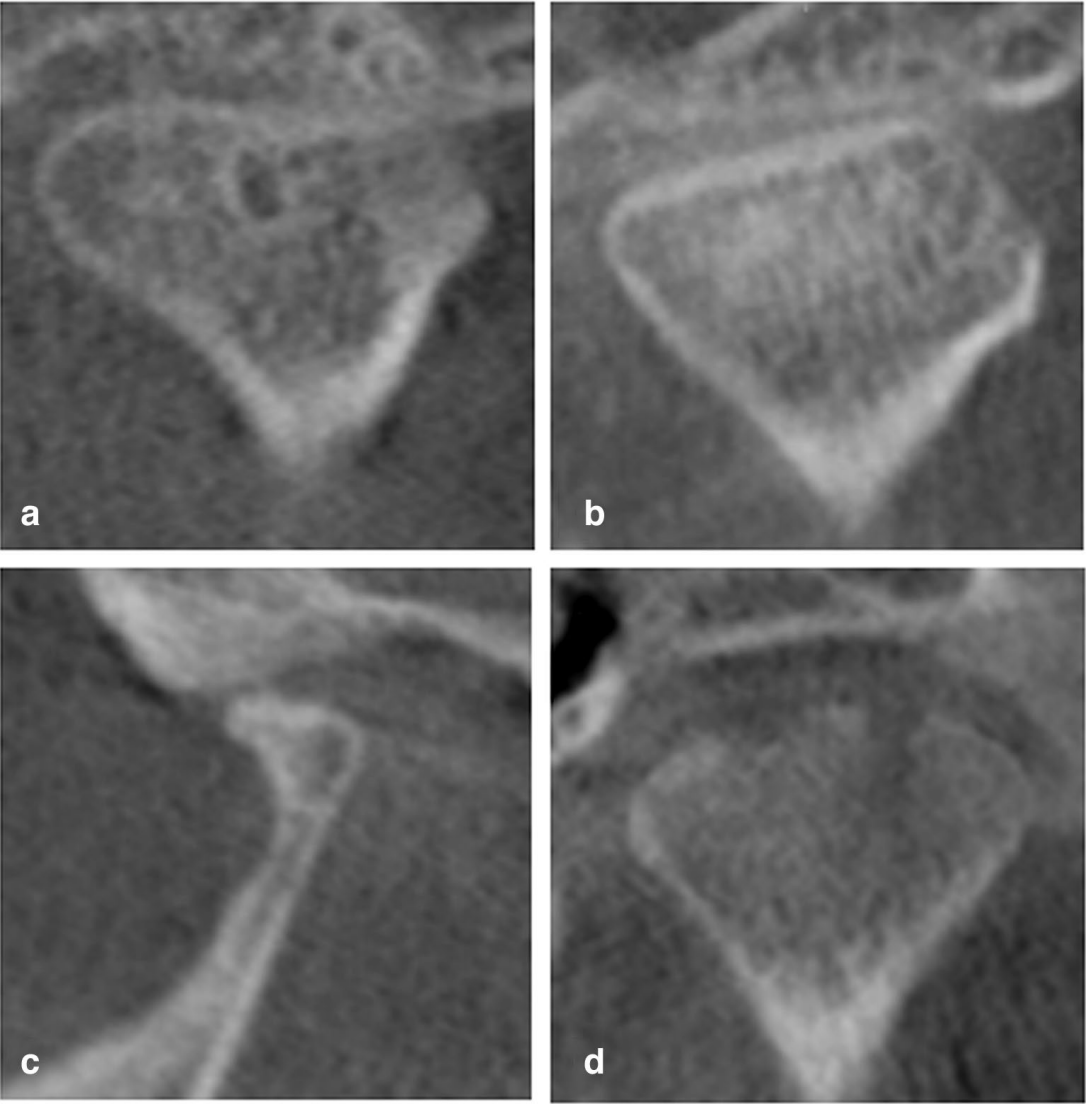
The radiographic criteria used to confirm the diagnosis of osteoarthritis. (**a**) Subcortical cyst (radiolucent area that may be just below the cortex or in the trabecular bone). (**b**) Flattening of the articular surfaces (disappearance of the even convexity or concavity of the articular surfaces). (**c**) Osteophyte (bony outgrowth arising from a mineralized joint surface). (**d**) Surface erosion (condylar surface irregularities).

**Figure 2:**
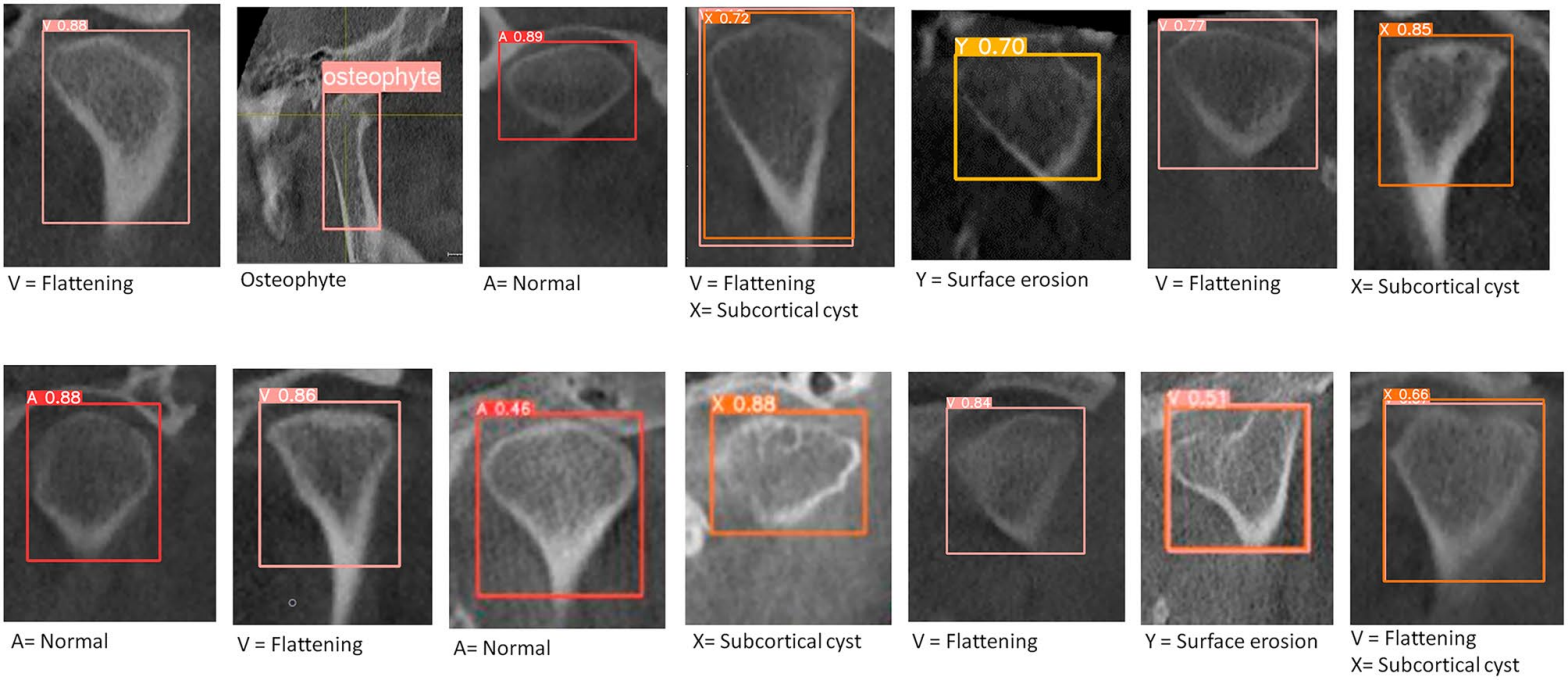
Sample CBCT images used in the validation of the AI model.

The study was approved ethically by the Institutional Review Board at the University of Sharjah (REC-20-09-21-01). The ethical principles for medical research involving human subjects as mentioned in the Declaration of Helsinki and the “Strengthening the Reporting of Observational studies in Epidemiology” (STROBE)^20^ guidelines were applied in this study. Patients aged 18–80 years who visited the Oral Diagnosis and Urgent Care Clinic at the University Dental Hospital Sharjah from November 2020 to November 2022 were enrolled in the study and divided into two groups; the TMJ Osteoarthritis and Control groups, respectively. TMJ clinical examination was done for all subjects as part of the initial screening. All subjects were informed about the aim of the study and were requested to sign consent forms before participation. The inclusion criteria for the TMJ Osteoarthritis group involved all the signs and symptoms of osteoarthritis as crepitus, joint pain in the last 30 days, deviation on mouth opening and limitation in opening. Patients were evaluated using the DC/TMD Axis-I assessment instruments as pain screener^21^, symptom questionnaire^22^ and demographic questionnaire^22^ and Axis-II assessment instruments as pain drawings, Graded Chronic Pain Scale^23^, disability score^21^ and Jaw Functional Limitation Scale^21^. Only patients with acute or serious dysfunction symptoms according to Helkimo’s clinical dysfunction index (Di)^24^ were entitled for CBCT exposure. All subjects in the Control group were assessed for the absence of all the clinical and radiographic signs and symptoms of TMD and were recruited when they had CBCT records for reasons other than TMD. Pregnant women, patients who had past TMD treatment and patients who had systemic conditions that could cause joint deformity were excluded. The initial screening procedures were done by a clinical tutor and the DC/TMD assessment was done by an oral and maxillofacial surgeon (W.T.) with more than 20 years’ experience in the management of TMD and an oral radiologist (S.A.) with more than 25 years’ experience and both were blinded to the results of the initial screening. The evaluators were calibrated prior to the start of the study by examining the same 20 patients independently following the DC/TMD protocol. The CBCT examinations were conducted using GALILEOS 3-D X-ray systems (SIRONA Dental Systems, York, PA). The CBCT protocol was: tube voltage: 85 kV, scanning time: 14 s, tube current: 7 mA, effective dose: 75 mSv, voxel size: 150 mm (screen resolution 1366 × 768), field of view: 15 × 15 cm, and slice thickness 1.0 mm. The TMJs were evaluated from the medial pole to the lateral pole in sagittal, coronal and axial planes. The radiographic criteria that were used to confirm the diagnosis of osteoarthritis were subcortical cyst (radiolucent area that may be just below the cortex or in the trabecular bone) (Fig. 1a), flattening of the articular surfaces (disappearance of the even convexity or concavity of the articular surfaces) (Fig. 1b), osteophyte (bony outgrowth arising from a mineralized joint surface) (Fig. 1c) and surface erosion (condylar surface irregularities) (Fig. 1d)^25^. The CBCT radiographs were assessed twice on a 1-week interval by 2 evaluators (W.T. and S.A.) independently. The 2 evaluators then reassessed the records in which there was a disagreement collectively on a third occasion to reach consensus. To ensure a strict blinding protocol, two other researchers (A.K. and S.T.) were responsible for the data entry, training, and testing of the AI model. A total of 2737 images, saved as TIFF files, were used for the training and validation of the AI model, including 1986 cross-sectional images and 751 tangential images. The images used for training and validation were selected from the CBCT records of 943 patients who had 792 osteoarthritic joints and 1094 normal joints (Fig. 2). The images were rescaled to 640 × 640 pixels, and the area of interest was marked in a rectangle and converted to XY coordinates using the LabelImg software (Tzutalin. LabelImg. Git code 2015). The cross-sectional images were used to detect flattening, subcortical cyst and surface erosion, whereas the tangential images were used to detect osteophytes.

### Development, training and testing of the AI model

**Figure 3:**
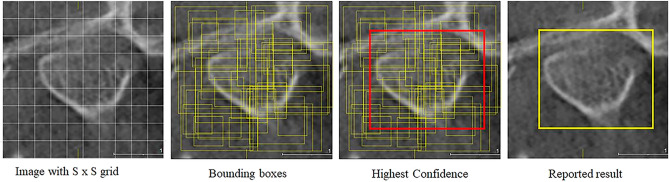
Object detection was achieved using a single regression model from the pixels to the coordinates of the bounding boxes and the class probabilities The input image is divided into an S × S grid. If the center of an object falls into a grid cell, that grid cell is responsible for detecting that object. Each grid cell predicts bounding boxes and confidence scores for those boxes.

Our model was based on the You Only Look Once (YOLO) system that uses a single convolutional network to concurrently predict several bounding boxes and generate the class probabilities^26,27^. YOLO achieves object detection using a single regression model from the pixels to the coordinates of the bounding boxes and the class probabilities. The model trains on full images and directly optimizes the detection performance. During object detection, the input image is divided into an S × S grid. If the center of an object falls into a grid cell, that grid cell is responsible for detecting that object. Each grid cell predicts bounding boxes and confidence scores for those boxes (Fig. 3). The YOLO model used in the present study was YOLO5v7 Large with 477 layers, 76,185,580 parameters, 76,185,580 gradients, 110.5 billion floating-point operations per second (GFLOPs). Optimization of the model used the Stochastic Gradient Descent (SGD) with parameter groups 131 weight (decay = 0.0), 135 weight (decay = 0.0005) and 135 bias. The model environment was composed of a central processing unit (CPU) with 12th Generation Intel(R) Core i9-12900K, 3.19 GHz, 128 GB RAM and NVIDIA RTX 3090 Ti, 24,564 GB. The network has convolutional layers that recognize features of the image followed by totally connected layers that undergo prediction of the probabilities^27,28^. Alternating 1 × 1 convolutional layers reduce the features space from preceding layers. Training of the network was performed for about 135 epochs on the training and validation datasets using a batch size of 64 using Python 3.9.16, PyTorch 1.13.1 and CUDA 11.7. The training hyperparameters were: learning rate = 0.01, momentum = 0.937, weight decay = 0.0005, warmup epochs = 3.0, warmup momentum = 0.8, warmup bias learning rate = 0.1, box = 0.05, cumulative layout shift (cls) = 0.5, cls_poisitive weight = 1.0, objectness loss gain (obj) = 1.0, objectness positive weight = 1.0, training intersection over union (IoU) threshold = 0.5. The learning rate schedule was 10^−3^ for the first epochs then raised to 10^−2^ for 75 epochs, then 10^−3^ for 30 epochs then 10^−4^ for the last 30 epochs. A completely separate testing set composed of 350 images (250 cross-sectional images and 100 tangential images) was used for testing the model. The set had a single image for each TMJ of 175 patients.

During the testing phase, two experienced evaluators (W.T. and S.A.) performed a DC/TMD-based assessment, including the evaluation of the CBCT records, on the 350 patients constituting the testing set. The CBCT radiographs were assessed twice on a 1-week interval by the 2 evaluators independently and reassessed in case of disagreement collectively on a third occasion to reach consensus. The concluded diagnosis generated from this assessment was considered as the golden reference. The performance of the AI model in diagnosing osteoarthritis from the CBCT images was then compared to an oral radiologist (S.S.) against the golden reference. The oral radiologist had 15 years of clinical experience and was blinded to the research protocol.

The diagnostic performance measures used for the assessment of the diagnostic performance of the AI model were sensitivity, specificity, positive predictive values (PPV), negative predictive values (NPV), intersection over union between detected object and ground truth polygon (IoU), test accuracy and the Kappa coefficient of agreement. Sensitivity is defined as the proportion of true positive results to all positive results (Sensitivity = true positives/(true positives + false negatives)). Specificity is the proportion of true negative results to all negative results (Specificity = true negatives/(true negatives + false positives)). PPV is the proportion of positive test results which are actually positive, according to the golden reference, to the total positive test results (PPV = true positives/(true positives + false positives)). NPV is the proportion of negative test results which are actually negative, according to the golden reference, to the total negative test results (NPV = true negatives/(true negatives + false negatives)). IoU is the ratio of the overlapping between the predicted bounding box and the ground truth bounding box. Test accuracy is the proportion of all the true test results to all test results (Test Accuracy = (true positives + true negatives)/(sum of All results)).

### Statistical analysis

Statistical analysis was performed by SPSS (version 20). Percentages of agreement were compared using *Z* test of two proportions. The level of significance was set at P < 0.05. Two Tailed tests were assumed throughout the analysis for all statistical tests.

## Results

According to the DC/TMD-based golden reference, the testing set had 73 condylar flattening, 134 subcortical cysts, 11 surface erosions and 38 osteophytes. The performance measures of the AI model are shown in Table 1. The diagnostic performance measures of the AI model and the oral radiologist against the golden reference are shown in Tables 2 and 3 respectively. The AI diagnosis showed statistically higher agreement with the golden reference compared to the oral radiologist. Cohen’s kappa showed statistically significant differences in the agreement between the AI and the oral radiologist with the golden reference for the diagnosis of all signs collectively (*P* = 0.0079) and for the diagnosis of subcortical cysts (*P* = 0.0214). The differences in agreement were not significant for the diagnosis of condylar flattening (*P* = 0.0595), surface erosion (*P* = 0.1581) and osteophyte (*P* = 0.3886). The osteophytes had the lowest agreement whereas the surface erosions showed the highest agreement with the golden reference. Both the AI model and the oral radiologist showed high test accuracy (> 0.95), however the AI model showed equal or better test accuracy compared to the oral radiologist (Table 4). The agreement between the AI model diagnosis and the oral radiologist diagnosis is shown in Table 5.

**Table 1:** The performance measures of the AI model.

PPV (95% confidence limits)	Sensitivity (95% confidence limits)	Test accuracy (95% confidence limits)	mAP50%	IoU	Total training time
0.96 (0.93–0.98)	0.98 (0.96–1.00)	0.99 (0.98–0.99)	94%	0.9308	5 h

*PPV* positive predictive values, *mAP* mean average precision, *IoU* intersection over union.

**Table 2:** The diagnostic performance of the AI diagnosis against the golden reference.

	Diagnostic performance					Cohen’s kappa	Kappa index	P value
	Sensitivity (95% confidence limits)	Specificity (95% confidence limits)	PPV (95% confidence limits)	NPV (95% confidence limits)	Test accuracy (95% confidence limits)			
Condylar flattening	0.96 (0.91–1.00)	1.00 (1.00–1.00)	1.00 (1.00–1.00)	0.99 (0.98–1.00)	0.99 (0.98–1.00)	0.97	Near perfect agreement	0.0000**
Subcortical cyst	0.99 (0.98–1.00)	0.98 (0.96–1.00)	0.96 (0.93–0.99)	1.00 (0.99–1.00)	0.98 (0.97–1.00)	0.96	Near perfect agreement	0.0000**
Surface erosion	1.00 (1.00–1.00)	1.00 (1.00–1.00)	1.00 (1.00–1.00)	1.00 (1.00–1.00)	1.00 (1.00–1.00)	1.00	Perfect agreement	0.0000**
Osteophyte	0.97 (0.92–1.00)	0.98 (0.97–1.00)	0.86 (0.76–0.96)	1.00 (0.99–1.00)	0.98 (0.97–0.99)	0.90	Near perfect agreement	0.0000**
All signs	0.98 (0.96–1.00)	0.99 (0.98–1.00)	0.96 (0.93–0.98)	1.00 (0.99–1.00)	0.99 (0.98–0.99)	0.96	Near perfect agreement	0.0000**

**Statistically highly significant.

*PPV* positive predictive values, *NPV* negative predictive values.

**Table 3:** The diagnostic performance of the oral radiologist against the golden reference.

	Diagnostic performance					Cohen’s kappa	Kappa index	P value
	Sensitivity (95% confidence limits)	Specificity (95% confidence limits)	PPV (95% confidence limits)	NPV (95% confidence limits)	Test accuracy (95% confidence limits)			
Condylar flattening	0.89 (0.82–0.96)	1.00 (1.00–1.00)	1.00 (1.00–1.00)	0.97 (0.95–0.99)	0.98 (0.96–0.99)	0.93	Near perfect agreement	0.0000**
Subcortical cyst	0.93 (0.88–0.97)	0.98 (0.96–1.00)	0.96 (0.93–0.99)	0.95 (0.93–0.98)	0.96 (0.94–0.98)	0.91	Near perfect agreement	0.0000**
Surface erosion	0.91 (0.74–1.00)	1.00 (1.00–1.00)	1.00 (1.00–1.00)	1.00 (0.99–1.00)	1.00 (0.99–1.00)	0.96	Near perfect agreement	0.0000**
Osteophyte	0.95 (0.88–1.00)	0.98 (0.97–1.00)	0.86 (0.75–0.96)	0.99 (0.98–1.00)	0.98 (0.96–0.99)	0.89	Near perfect agreement	0.0000**
All signs	0.92 (0.88–0.95)	0.99 (0.98–1.00)	0.96 (0.93–0.98)	0.98 (0.97–0.99)	0.98 (0.97–0.98)	0.92	Near perfect agreement	0.0000**

**Statistically highly significant.

*PPV* positive predictive values, *NPV* negative predictive values.

**Table 4:** Test accuracy of the AI model compared to the oral radiologist.

	AI model	Oral radiologist	z	P value
Condylar flattening	0.99	0.98	1.52	0.12863
Subcortical cyst	0.98	0.96	1.99	0.04614*
Surface erosion	1.00	1.00	Equal values	
Osteophyte	0.98	0.98	0.26	0.79408
All signs	0.99	0.98	2.33	0.01984*

*Statistically significant.

**Table 5:** The agreement between the AI model diagnosis and the oral radiologist diagnosis.

	Percentage of agreement (95% confidence limits)	Cohen’s kappa	P value
Condylar flattening	98.57% (97.33–99.81%)	0.95	0.0000**
Subcortical cyst	97.43% (95.77–99.09%)	0.95	0.0000**
Surface erosion	99.71% (99.16–100.00%)	0.96	0.0000**
Osteophyte	99.71% (99.16–100.00%)	0.99	0.0000**
All signs	98.86% (98.30–99.41%)	0.96	0.0000**

**Statistically highly significant.

## Discussion

Osteoarthritis of the TMJ comprises complex pathophysiological processes and necessitates comprehensive evaluations to detect the remodeling and degeneration of the bone and cartilage. Increasing attention has now focused on the early diagnosis of osteoarthritis to stop the disease progression and prevent or decrease the permanent joint damage^3^. The clinical diagnosis of TMJ osteoarthritis relies on the radiographic features of the joint components that involve early signs of osseous remodeling like flattening and sclerotic changes followed by erosive, attrition, osteophytes and cyst-like lesions. The diagnostic accuracy, safety and reliability of CBCT in detecting the bony changes associated with osteoarthritis have been reported to be superior to other imaging modalities^25,29^. The primary diagnosis of TMD has been changed after the CBCT assessment in 26.08% of cases in one study^25^ and in 58% of cases in another study^30^. Several factors may hinder the early diagnosis of osteoarthritis and these include the variability in the examination methods, diagnostic criteria and taxonomy between different clinical and research centers^8^. Among these factors is the subjectivity of the radiographic diagnosis of the disorder. The inconsistency in the diagnosis of osteoarthritis was evident in one study that showed that 206 patients consulted an average of 30 providers from 44 different specialties to seek treatment for their TMD^31^. Another study showed that 101 orofacial pain patients had multiple consultations with 15 different specialties to diagnose and treat their pain^32^. The urgent need to eliminate the subjectivity in the interpretation of the radiographic signs of osteoarthritis was emphasized in several studies to facilitate and expedite the diagnosis of TMJ osteoarthritis^17,33^. Thus, an automated and precise diagnostic system is warranted to improve the accuracy of the diagnosis of TMJ osteoarthritis.

AI aims to match and surpass the cognitive abilities of humans and secure a new era of improved standards of care in medicine. Date-driven AI or deep learning utilizes the data generated by humans to train mathematical models and build a cognitive system that is able to analyze and reach a diagnosis. Neural networks are a subset of deep learning that is inspired by the nervous system and contain layers of interconnected neurons. Neural networks transfer signals between the different layers of neurons through a convolutional process to learn the features within the data^34^. Object detection systems in general starts by detecting the potent features in the input images, then use classifiers to identify the objects in space. The classifiers are applied over a sliding window on the image or on particular areas of the image^35^. In the present study, the AI model was based on YOLO; a detector that is trained to detect a diversity of objects simultaneously. YOLO puts structural constrains on the grid cell proposals which support multiple simultaneous recognitions of the same object. The system suggests only 98 bounding boxes for one image compared to 2000 for other object recognition systems then incorporates the individual components into a single rectified model^26,27^. The results have shown that the diagnostic performance measures of the AI model were in statistically higher agreement with the golden reference compared to the oral radiologist. Both the AI model and the oral radiologist had high test accuracy over 0.95, however the AI model had always equal or better test accuracy compared to the oral radiologist. Similar results were obtained in a study that compared the performance of dental clinicians and a multilayer perceptron neural network in diagnosis of orofacial pain and TMD and found that the performance of the neural network was significantly higher than the dental clinicians^36^. Another study used the Karas’ ResNet AI model for the radiographic diagnosis of TMJ osteoarthritis from orthopantomograms and compared the diagnostic performance of the model with an oral radiologist^11^. Their model lacked in performance in one the categories of TMJ osteoarthritis, however their model exhibited equal sensitivity to that of the oral radiologist and better balance between sensitivity and specificity. A Neural network was used in another study to classify the condylar morphology on CBCT records and evaluate the correlations between groups of biomarkers in patients with TMJ osteoarthritis^37^. The neural network staging of osteoarthritis as compared to the clinicians’ consensuses had a 91.2% accuracy, which is in accordance with the results of the present study. A natural language processing-based model utilizing artificial neural networks was able to diagnose TMD according to the analysis of the patient’s chief complaint and the measurement of the maximal mouth opening^38^. In the present study, the high test accuracy achieved by the AI model and the excellent agreements between the model and the radiologist can be attributed to several factors. Firstly, the criteria used for the radiographic diagnosis of osteoarthritis from CBCT records were based on the DC/TMD that was reported to have excellent sensitivity, specificity and interexaminer reliability and is considered the most universally accepted standardized protocol^14^. Secondly, the training database used in the present study was relatively increased compared to several previous studies. Finally, the AI model was trained to learn the detection of the radiographic signs that appear on the cross-sectional CBCT views independently of the training used to learn the signs on the tangential CBCT views.

The performance of object detectors is commonly challenged by several factors as the different resolution input compared to that used in training, foreground or background imbalance, smaller sized objects, low computational resources, small datasets and imprecise localizations during predictions. In general, the detection accuracy of two-stage object detectors exceed that of single-stage detectors, however with the development of the single-staged YOLO, its accuracy has surpassed several two-staged detectors^27^. YOLO has several advantages over other detection systems. Firstly, as YOLO is built as a single regression problem, it does not require a complex structure like other models and accordingly it is much faster. The YOLO network operates at a speed of 45 frames per second and the fast version at 150 frames per second. The network concomitantly predicts the bounding boxes, extracts the features and performs the circumstantial reasoning. Secondly, YOLO accomplishes at least double the mean average precision of the other object detection systems. Thirdly, YOLO analyzes the whole image while making detections and does not depend on region proposals and sliding windows like other systems. YOLO is trained on generalized presentations of objects so it generates less than half the average number of background errors compared to other systems^27^. In a recent study, YOLO-V3 has been compared to two other object detection algorithms; Single Shot Detection and Faster Region based Convolutional Neural Networks. The overall performance of YOLO-V3 was superior to the other two algorithms. YOLO-V3 was the fastest while maintaining excellent accuracy^39^. The main limitation of YOLO is the difficulty of the detection of objects in new and unfamiliar aspect ratios or presentation, however this limitation is not applicable to the diagnosis of osteoarthritis from CBCT records^27^.

The timely identification of signs of TMJ osteoarthritis and the detection of patients at risk of progression to advanced forms may facilitate the development of more effective treatment approaches. This requires the progression of the current binary classifications to multinomial regression functions that recognize the clinical and radiographic findings in addition to the different biomarkers and classify the early, moderate and severe forms of TMJ osteoarthritis. The development of multinomial deep learning classifiers in the field of TMJ osteoarthritis can promote a line of research that investigates the possibility of diagnosis at an early reversible phase^40^. The present study validated an AI model that diagnosed the early and late radiographic signs of TMJ osteoarthritis and can be considered a base for further research to build such multinomial deep learning models.

## Conclusion

The AI model used in the present study had equal or better diagnostic performance for TMJ osteoarthritis compared to the human expert. The adoption of AI in the radiographic diagnosis of osteoarthritis is expected to eliminate the subjectivity associated with the human interpretation and expedite the diagnostic process thus reducing the probability of the disease progression. Further studies are required to refine the network capabilities and develop a multimodal cognitive system that analyzes the patients’ history, radiographic and clinical examination data to diagnose osteoarthritis and other forms of TMD efficiently.

## Data Availability

The data that support the findings of this study are available from the authors upon request.
